# Treatment satisfaction with molidustat in CKD-related anemia in non-dialysis patients: a post-hoc analysis of two clinical trials

**DOI:** 10.1007/s10157-023-02353-x

**Published:** 2023-04-24

**Authors:** Hiroyasu Yamamoto, Takashi Yamada, Ken Miyazaki, Takuto Yamashita, Takuya Kato, Kenichi Ohara, Yusuke Nakamura, Tadao Akizawa

**Affiliations:** 1 Division of Nephrology and Hypertension, Department of Internal Medicine, The Jikei University School of Medicine, 3-25-8 Nishi-Shimbashi, Minato-Ku, Tokyo, 105-8461 Japan; 2 Research and Development, Bayer Yakuhin Ltd, 2-4-9 Umeda, Kita-Ku, Osaka, 530-0001 Japan; 3 Market Access, Bayer Yakuhin Ltd, Marunouchi Kitaguchi Bldg. 1-6-5, Marunouchi, Chiyoda-Ku, Tokyo, 100-8265 Japan; 4 Showa University School of Medicine, 1-5-8 Hatanodai, Shinagawa-Ku, Tokyo, 142-8555 Japan

**Keywords:** Renal anemia, Clinical trials, CKD (chronic kidney disease), Treatment satisfaction, Molidustat

## Abstract

**Background:**

Erythropoiesis-stimulating agents (ESAs) are the standard treatment for patients with renal anemia to increase hemoglobin (Hb) levels and reduce the need for blood transfusions. However, treatments targeting high Hb levels require high doses of ESAs administered intravenously, which is associated with an elevated risk of adverse cardiovascular events. Furthermore, there have been some problems such as hemoglobin variability and low achievement of target hemoglobin due to the shorter half-lives of ESAs. Consequently, erythropoietin-promoting medications, such as hypoxia-inducible factor-prolyl hydroxylase (HIF-PH) inhibitors, have been developed. This study aimed to evaluate changes in the Treatment Satisfaction Questionnaire for Medicine version II (TSQM-II) domain scores relative to baseline in each trial, to assess patient satisfaction with molidustat versus darbepoetin alfa.

**Methods:**

This post-hoc analysis of two clinical trials compared treatment satisfaction with an HIF-PH inhibitor, molidustat, versus a standard ESA, darbepoetin alfa, as part of therapy in patients with non-dialysis chronic kidney disease (CKD) and renal anemia.

**Results:**

Exploratory outcome data using the TSQM-II showed that both arms in both trials had enhanced treatment satisfaction over the course of the study period, as well as improvements in most TSQM-II domains at week 24 of treatment. Molidustat was associated with convenience domain scores at multiple time points depending on the trial. More patients were highly satisfied with the convenience of molidustat than that of darbepoetin alfa. Patients treated with molidustat had increased global satisfaction domain scores compared with those treated with darbepoetin alfa; however, the differences in global satisfaction domain scores were not significant.

**Conclusion:**

These patient-reported satisfaction outcomes support the use of molidustat as a patient-centered treatment option for CKD-related anemia.

**Registration of clinical trials:**

ClinicalTrials.gov Identifier: NCT03350321 (November 22, 2017).

ClinicalTrials.gov Identifier: NCT03350347 (November 22, 2017).

**Supplementary Information:**

The online version contains supplementary material available at 10.1007/s10157-023-02353-x.

## Introduction

Chronic kidney disease (CKD) is a progressive illness defined by impaired kidney function, as measured using the estimated glomerular filtration rate (eGFR), albuminuria thresholds, or kidney injury duration [1]. The global prevalence of CKD was approximately 13% in 2016, which is thought to be increasing and partly attributable to societal aging [2]. In Japan, the age-adjusted prevalence of CKD has increased from 57.8 per 1000 adults in 2005 to 71.8 per 1000 adults in 2017, according to a real-world database study of the general population [3]. Furthermore, many patients with CKD remain undiagnosed or underdiagnosed in Japan [4, 5], suggesting that the burden of CKD is poorly recognized.

Erythropoietin synthesis is directly affected by impaired kidney function in patients with CKD, which limits hemoglobin (Hb) production, leading to serious complications such as anemia. The average prevalence of anemia is approximately twice as high in patients with CKD than in the general population [6], and its incidence increases rapidly as the disease progresses [7]. CKD-related anemia is associated with a lower health-related quality of life (HRQoL) than that in CKD patients without anemia [8]. The standard treatment for patients with CKD-related anemia encompasses increasing Hb levels using erythropoiesis-stimulating agents (ESAs) [9]. Although long-acting ESAs have been developed that ameliorate the problems of hemoglobin variability and low achievement of target hemoglobin due to their relatively short half-lives, but treatment methods requiring high doses of ESAs are associated with a risk of adverse cardiovascular events [10]. In addition to finding safe and efficacious alternatives to ESAs, oral treatments that are favorably received by patients are crucial for ensuring patient-centered care during the long-term treatment required for non-dialysis CKD-related anemia [11].

Two randomized, parallel-group, open-label, multicenter trials in Japan have shown that molidustat, a novel inhibitor of hypoxia-inducible factor-prolyl hydroxylase (HIF-PH), is efficacious and safe for treating non-dialysis CKD anemia as an alternative to a conventional ESA such as darbepoetin alfa [12, 13]. The phase III trials entitled Molidustat once dailY improves renal Anaemia By Inducing EPO (MIYABI) Non-Dialysis correction (ND-C) and MIYABI Non-Dialysis-Maintenance (ND-M) had similar designs and enrolled similar patient populations, apart from MIYABI ND-C, which included only patients naïve to previous ESA treatment. The primary outcome of these trials was changes in the hemoglobin (Hb) levels. The treatment satisfaction reported by patients with their assigned medication was also evaluated using the Treatment Satisfaction Questionnaire for Medicine version II (TSQM-II) as an exploratory endpoint during each trial.

This post-hoc analysis aimed to evaluate the changes in TSQM-II domain scores relative to baseline in each trial, to assess patient satisfaction with molidustat versus darbepoetin alfa.

## Methods

Our primary objective was to evaluate the treatment satisfaction with molidustat and darbepoetin alfa versus baseline in non-dialysis CKD patients with renal anemia. This post-hoc analysis employed patient characteristics and TSQM-II data generated in each trial. The MIYABI ND-C and MIYABI ND-M trials have been described in detail elsewhere [12, 13]. Briefly, each trial compared the efficacy and safety of oral molidustat versus subcutaneous (SC) darbepoetin alfa by randomizing 150 patients to receive one comparator for 52 weeks. The treatment groups in MIYABI ND-C were patients treated with molidustat (molidustat group) and those treated with darbepoetin alfa (darbepoetin group), whereas the treatment groups in MIYABI ND-M were patients who had received previous ESA treatment and were prospectively treated with molidustat or darbepoetin alfa.

Treatment satisfaction was analyzed separately in each trial for the purpose of this post-hoc analysis, as the patient populations differed slightly between the two trials. However, the variables and statistical analysis for patient characteristics and outcomes from both trials were the same. The descriptive patient baseline characteristics of interest were age, sex, body mass index, smoking history, alcohol consumption, Hb level, medical history, history of thromboembolic events, estimated glomerular filtration rate (eGFR) category (mL/min/1.73 m^2^) (< 15, 15 to < 30, ≥ 30), previous ESA dose (MIYABI ND-M only), severity of CKD (modified KDIGO CKD guideline [14]), and risk of disease progression [14, 15].

Continuous variables are expressed as the number of non-missing values, median, mean, and standard deviation. Frequency tables were generated for the categorical variables.

**Fig. 1:**
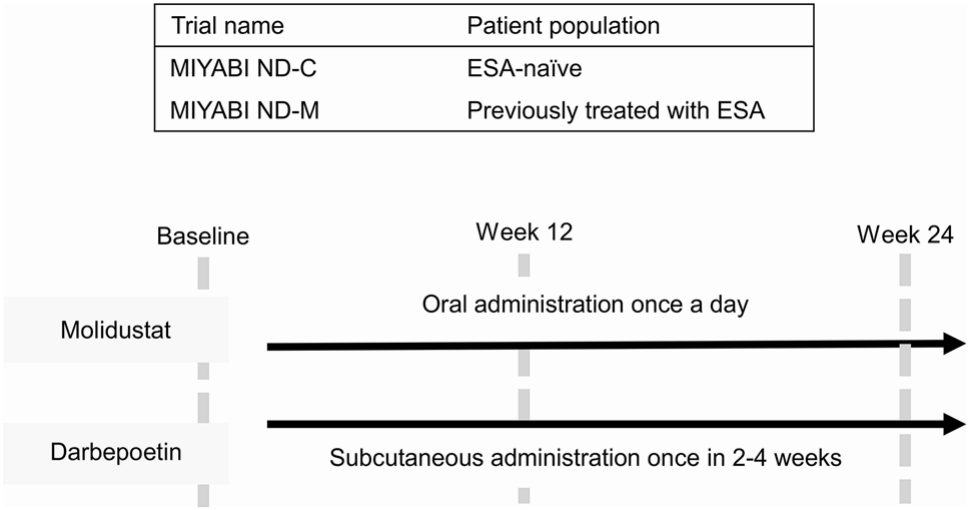
Overall study design for TSQM-II evaluation in both trials. *TSQM-II* Treatment Satisfaction Questionnaire for Medicine version II

In both trials, patients who had undergone study treatment at least once were asked to complete the TSQM-II at baseline, week 12, and week 24 (Fig. 1). The TSQM-II is an 11-item, self-administered, psychometrically valid measure of patient-reported satisfaction with medication [16] and has demonstrated validity and reliability among Japanese patients with chronic diseases [17]. The TSQM-II comprises the following four domains: effectiveness, side effects, convenience, and global satisfaction. Items 4, 5, and 6 each have five response options that range from “Extremely Dissatisfied” (1) to “Not at All Dissatisfied” (5), and all other items are scored using a 7-point response option scale ranging from “Extremely Dissatisfied” (1) to “Extremely Satisfied” (7). The total domain scores ranged from 0 to 100, with higher scores indicating better outcomes for the respective domains. In each trial, the scores for each domain were calculated according to the developer’s recommendations [18], and individual patient domain scores were computed if no more than one response item was missing from the domain. Patients who scored in the highest quartile for the global satisfaction domain from the entire study population at each visit were defined as having high satisfaction. Domain scores were described at each time point using summary statistics, and the mean change was calculated between baseline and other time points. The TSQM-II analysis dataset consisted of the subjects who received at least one study treatment dose. To evaluate change in scores, these subjects had a valid TSQM-II score at baseline as well as, at least one other time point.

Changes in the scores from baseline to each time point were compared between the molidustat group and darbepoetin group. The changes from baseline were assessed with paired t-tests. The least square (LS) mean change was estimated using a general linear model (GLM) controlling for baseline TSQM scores, age, sex, CKD duration, duration of anemia due to CKD, CKD etiology, eGFR category, risk of CKD progression, and Hb level. The effect of treatment with molidustat versus darbepoetin alfa was assessed for each questionnaire domain using a generalized estimating equation (GEE) model adjusting for imbalances at baseline. Given the expected correlations between assessments, an autoregressive correlation structure is specified. The covariates in the GEE model were treatment, time, the interaction between time and treatment, and imbalanced variables at baseline that were clinically important. Finally, Pearson correlation coefficients were calculated for the relationship between Hb levels and TSQM scores among patients with high satisfaction at week 24, as well as between the average drug dose (from baseline to week 24) and TSQM scores (at week 24). All analyses were conducted for exploratory purposes, with no multiplicity adjustment for statistical tests.

## Results

### MIYABI ND-C

**Fig. 2:**
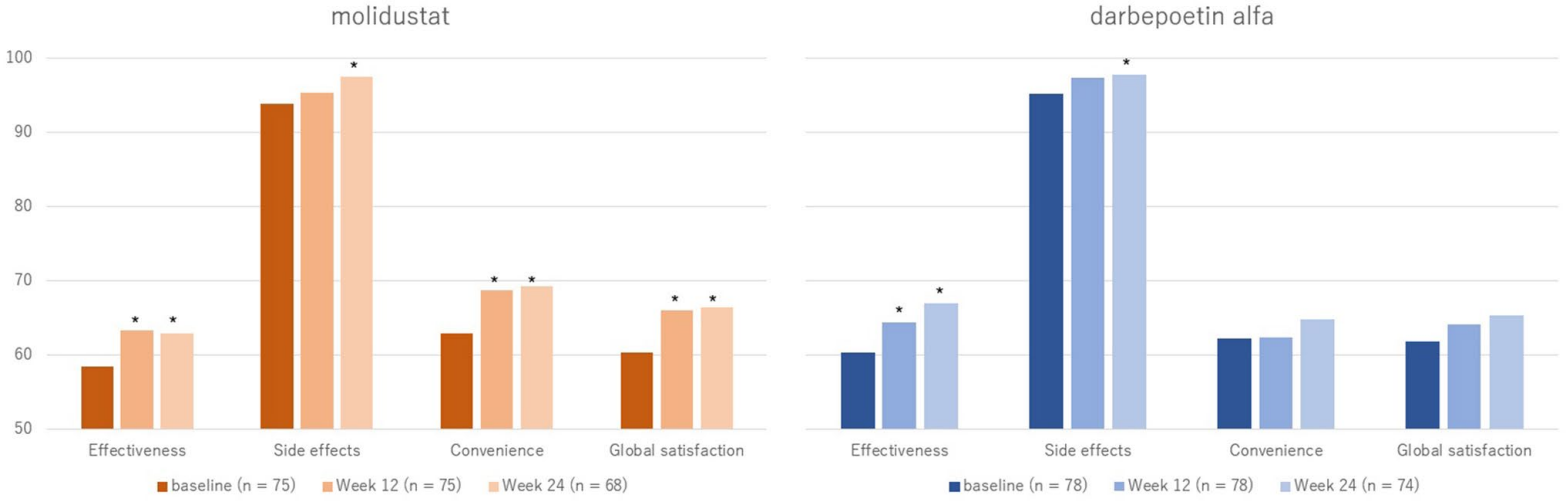
TSQM-II mean domain scores for MIYABI ND-C. Bold indicates significant (*p* < 0.05) improvement at the evaluation timepoint compared to baseline. *TSQM-II* Treatment Satisfaction Questionnaire for Medicine version II, *MIYABI* molidustat once daily improved renal anemia by inducing EPO, *ND-C* non-dialysis correction

A total of 153 patients were included in the analysis of the MIYABI ND-C trial (75 for the molidustat group and 78 for the darbepoetin group). In the molidustat group, 96.0% of patients were categorized as having a very high risk for CKD progression, while 83.3% of patients in the darbepoetin group were categorized as having a very high risk. Other baseline characteristics were similar between the two groups (Table 1). Patients in the molidustat and darbepoetin groups reported a nominally significant improvement in effectiveness domain scores at 12 and 24 weeks (*p* < 0.05). The mean $\pm$ standard deviation (SD) changes from baseline in effectiveness domain scores were 4.9 ± 14.0 at week 12 and 3.9 ± 15.2 at week 24 in the molidustat group. However, in the darbepoetin group, the mean changes in effectiveness domain scores increased progressively from baseline to week 12 (4.1 ± 16.5) and from baseline to week 24 (6.8 ± 17.8). Significant improvements in the convenience and global satisfaction domains were also observed only for the molidustat group at weeks 12 and 24 (*p* < 0.01), and the mean scores for the side effect domain were 95.2 (median: 100) and 97.4 (median: 100) at weeks 12 and 24, respectively (Fig. 2). Higher side effect domain scores indicated that patients were less bothered by the side effects.

**Table 1:** Baseline patient characteristics in the MIYABI ND-C trial

Characteristics	ND-C	
	Molidustat (*n* = 75)	Darbepoetin alfa (*n* = 78)
Age, mean (SD), year	71.79 (9.34)	71.08 (10.11)
Age group, *n* (%)		
< 65	15 (20.0)	18 (23.1)
65 to < 75	27 (36.0)	28 (35.9)
75 ≥	33 (44.0)	32 (41.0)
Female: Male, *n* (%)	30:45 (40.0: 60.0)	29:49 (37.2: 62.8)
BMI, mean (SD), kg/m^2^	24.01 (3.45)	23.99 (3.45)
Hb level, mean (SD), g/dL	9.83 (0.65)	10.01 (0.60)
Duration of CKD, mean (SD), year	7.48 (7.66)	8.54 (10.71)
Duration of anemia due to CKD, mean (SD), year	2.47 (2.81)	3.26 (3.08)
Etiology of CKD, *n* (%)		
Diabetic nephropathy	29 (38.7)	20 (25.6)
Others	42 (56.0)	56 (71.8)
Unknown	4 (5.3)	2 (2.6)
History of thromboembolic events, *n* (%)	6 (8.0)	7 (9.0)
eGFR group (mL/min/1.73 m2), *n* (%)		
< 15	26 (34.7)	25 (32.1)
15 to < 30	39 (52.0)	34 (43.6)
30 +	10 (13.3)	19 (24.4)
Risk of progression of CKD, *n* (%)		
Moderately increased risk: G3aA1	1 (1.3)	2 (2.6)
High-risk: G3aA2, G3bA1	2 (2.7)	11 (14.1)
Very high-risk: G3aA3, G3bA2, G3bA3, G4A1, G4A2, G4A3, G5A1, G5A2, G5A3	72 (96.0)	65 (83.3)

Patients who had scores at baseline and at least one other timepoint for any of the domains were included

The mean change in LS from baseline to each time point showed that patients treated with molidustat showed a nominally significant improvement in the convenience domain at week 12 compared to patients taking darbepoetin alfa (Table 2); however, a numeric but insignificant difference was noted between the groups at week 24. In the GEE model, only the convenience domain was significantly different at week 12, with a significant interaction term between time and treatment (Table 3). Pearson correlation coefficients for the correlation between the TSQM-II domain scores and Hb level or average drug dose were not significantly related (Supplementary Table 1).

**Table 2:** Adjusted LS mean changes in TSQM-II scores for each domain at each timepoint and differences across treatment groups (MIYABI ND-C)

	ND-C			
	Molidustat	Darbepoetin alfa	Difference	*p*-value
Week 12: *n*, mean (SE)	75, 5.4 (3.0)	78, 4.4 (2.7)	1.0 (2.6)	0.703
Week 24: *n*, mean (SE)	68, 8.4 (3.4)	74, 9.9 (3.1)	− 1.5 (2.9)	0.609
Side effects				
Week 12: *n*, mean (SE)	75, 0.1 (2.9)	78, 2.0 (2.6)	− 2.0 (2.6)	0.440
Week 24: *n*, mean (SE)	68, 3.2 (2.7)	74, 2.3 (2.4)	0.9 (2.3)	0.696
Convenience				
Week 12: *n*, mean (SE)	75, 5.1 (3.2)	78, − 0.8 (2.9)	5.9 (2.8)	0.040*
Week 24: *n*, mean (SE)	68, 6.5 (3.6)	74, 2.2 (3.3)	4.3 (3.1)	0.167
Global satisfaction				
Week 12: *n*, mean (SE)	75, 3.0 (3.1)	78, − 0.1 (2.8)	3.1 (2.7)	0.266
Week 24: *n*, mean (SE)	68, 5.0 (3.4)	74, 2.2 (3.1)	2.9 (3.0)	0.344

Patients who had scores at baseline and at least one other time point for any of the domains were included

*p < 0.05

**Table 3:** GEE results for the impact of molidustat on changes in scores across treatment (MIYABI ND-C)

	ND-C			
	Effectiveness (*n* = 153)	Side effects (*n* = 153)	Convenience (*n* = 153)	Global satisfaction (*n* = 153)
	Coef (95% CI)	Coef (95% CI)	Coef (95% CI)	Coef (95% CI)
Timepoint*Treatment arm				
Week 12	0.83 (− 3.98, 5.63)	− 0.80 (− 5.59, 4.00)	5.49* (0.27, 10.71)	3.32 (− 1.75, 8.39)
Week 24	− 2.36 (− 7.64, 2.91)	0.86 (− 3.30, 5.03)	3.54(− 2.27, 9.35)	2.17 (− 3.28, 7.62)

Patients who had scores at baseline and at least one other timepoint for any of the domains were included. darbepoetin alfa was the reference treatment arm, and the baseline was the reference time point. Controls included the treatment arm, timepoint category, and baseline characteristics (age, sex, duration of CKD, duration of anemia due to CKD, main cause of CKD, eGFR category (15– < 30 mL/min/1.73 m^2^, ≥ 30 mL/min/1.73 m^2^), risk of CKD progression (very high-risk), and Hb level)

*p < 0.05

### MIYABI ND-M

In total, 157 patients were included in the MIYABI ND-M trial (79 in the molidustat group and 78 in the darbepoetin group). The darbepoetin group had a higher mean age (72.2 years) and a higher proportion of male patients (66.7%; Table 4). In the molidustat group, the largest proportion of patients had an eGFR < 15 (46.8%), whereas the largest proportion of patients receiving darbepoetin alfa (48.7%) had an eGFR of 15 to < 30. In total, 89.9% of patients in the molidustat group were classified as having a very high risk of CKD progression, whereas 97.4% of patients in the darbepoetin group were classified in the very high-risk group.

**Table 4:** Baseline patient characteristics in the MIYABI ND-M trial

Characteristics	ND-M	
	Molidustat (*n* = 79)	Darbepoetin alfa (*n* = 78)
Age, mean (SD), year	69.24 (10.04)	72.17 (9.83)
Age group, *n* (%)		
< 65	21 (26.6)	14 (18.0)
65 to < 75	32 (40.5)	26 (33.3)
75 ≥	26 (32.9)	38 (48.7)
Female: Male, *n* (%)	35: 44 (44.3: 55.7)	26: 52 (33.3: 66.7)
BMI, mean (SD), kg/m^2^	23.89 (3.70)	23.92 (4.27)
Hb level, mean (SD), g/dL	11.31 (0.69)	11.29 (0.63)
Duration of CKD, mean (SD), year	7.34 (7.33)	8.02 (8.60)
Duration of anemia due to CKD, mean (SD), year	3.26 (2.77)	3.08 (3.30)
Etiology of CKD, *n* (%)		
Diabetic nephropathy	28 (35.4)	22 (28.2)
Others	49 (62.0)	53 (68.0)
Unknown	2 (2.5)	3 (3.9)
History of thromboembolic events, *n* (%)	10 (12.7)	9 (11.5)
eGFR group (mL/min/1.73 m^2^), *n* (%)		
< 15	37 (46.8)	31 (39.7)
15 to < 30	29 (36.7)	38 (48.7)
30 +	13 (16.5)	9 (11.5)
Previous ESA, *n* (%)		
High	53 (67.1)	54 (69.2)
Low	26 (32.9)	24 (30.8)
Risk of progression of CKD, *n* (%)		
Moderately increased risk: G3aA1	1 (1.3)	0 (0.0)
High-risk: G3aA2, G3bA1	7 (8.9)	2 (2.6)
Very high-risk: G3aA3, G3bA2, G3bA3, G4A1, G4A2, G4A3, G5A1, G5A2, G5A3	71 (89.9)	76 (97.4)

Patients who had scores at baseline and at least one other time point for any of the domains were included

**Fig. 3:**
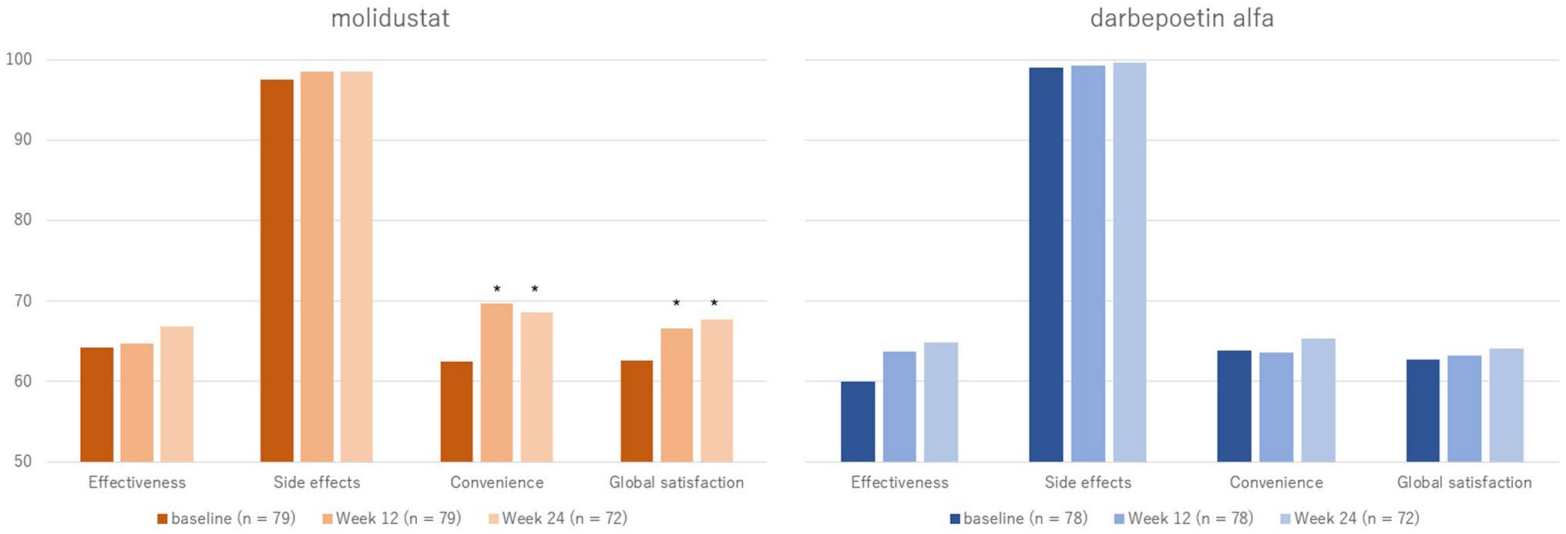
TSQM-II mean domain scores for MIYABI ND-M. *Indicates significant improvement at the evaluation timepoint compared to baseline. *TSQM-II* Treatment Satisfaction Questionnaire for Medicine version II, *MIYABI* molidustat once daily improved renal anemia by inducing EPO, *ND-C* non-dialysis correction

Patients who received molidustat reported a nominally significant improvement in the convenience and global satisfaction domain scores at 12 and 24 weeks from baseline (*p* < 0.05) (Fig. 3). The mean change scores for global satisfaction progressively improved from baseline to week 24 (increased by 3.6 at week 12 versus 5.3 at week 24). Significant changes were not observed in any domain at any follow-up time point for the darbepoetin group.

Similarly, no significant difference was noted in the mean change in LS in terms of the effectiveness, side effect, or global satisfaction domain scores between the treatment groups at each time point in the GLM model. However, patients treated with molidustat had nominally significant benefits in terms of convenience compared to those treated with darbepoetin alfa at weeks 12 and 24 (Table 5). The GEE model analysis for this trial showed that the convenience domain was significantly different at both time points, with a significant interaction term between time and treatment (*p* < 0.05) (Table 6). In the correlation analysis, the average drug dose significantly correlated with the effectiveness domain scores (Supplementary Table 2).

**Table 5:** Adjusted LS mean changes in TSQM-II scores for each domain at each timepoint and differences across treatment groups (MIYABI ND-M)

	ND-M			
	Molidustat	Darbepoetin alfa	Difference	*p*-value
Effectiveness				
Week 12: n, mean (SE)	79, 3.2 (3.1)	78, 5.8 (3.6)	− 2.6 (2.7)	0.347
Week 24: n, mean (SE)	72, 5.7 (3.2)	72, 7.7 (3.7)	− 2.0 (2.9)	0.490
Side effects				
Week 12: n, mean (SE)	79, − 0.9 (1.6)	78, − 1.9 (1.9)	1.0 (1.4)	0.480
Week 24: n, mean (SE)	72, − 0.9 (1.9)	72, − 1.7 (2.2)	0.8 (1.7)	0.624
Convenience				
Week 12: *n*, mean (SE)	79, 8.6 (2.9)	78, 1.7 (3.3)	6.9 (2.5)	0.006*
Week 24: *n*, mean (SE)	72, 6.3 (2.7)	72, 1.0 (3.2)	5.3 (2.4)	0.032*
Global satisfaction				
Week 12: *n*, mean (SE)	79, 3.5 (2.9)	78, 0.8 (3.4)	2.7 (2.6)	0.288
Week 24: *n*, mean (SE)	72, 5.1 (2.8)	72, 1.1 (3.3)	4.0 (2.5)	0.113

Patients who had scores at baseline and at least one other time point for any of the domains were included

^*^p < 0.05

**Table 6:** GEE results for impact of molidustat on changes in scores across treatment (MIYABI ND-M)

	ND-M			
	Effectiveness (*n* = 157)	Side effects (*n* = 157)	Convenience (*n* = 157)	Global satisfaction (*n* = 157)
	Coef (95% CI)	Coef (95% CI)	Coef (95% CI)	Coef (95% CI)
Timepoint*Treatment arm				
Week 12	− 3.21 (− 8.48, 2.05)	0.84 (− 1.79, 3.47)	7.53* (2.85, 12.20)	3.45 (− 1.27, 8.17)
Week 24	− 2.50 (− 7.85, 2.84)	0.44 (− 2.42, 3.29)	4.96* (0.47, 9.44)	3.92 (− 0.90, 8.75)

Patients who had scores at baseline and at least one other time point for any of the domains were included. darbepoetin alfa was the reference treatment arm, and the baseline was the reference time point. Controls included the treatment arm, timepoint category, and baseline characteristics (age, sex, duration of CKD, duration of anemia due to CKD, main cause of CKD, eGFR category (15– < 30 mL/min/1.73 m^2^, ≥ 30 mL/min/1.73 m^2^), risk of CKD progression (very high-risk), and Hb level)

*p < 0.05

## Discussion

The findings from this analysis suggest that when the results of both clinical trials are considered together, patients receiving molidustat reported improved treatment satisfaction over time. Patient-reported scores from both trials improved remarkably in terms of the convenience and global satisfaction domains when receiving molidustat. In contrast, the scores for darbepoetin alfa showed only numerical changes. Considering patient satisfaction in decision-making regarding CKD-related anemia treatment options may allow healthcare providers to better align care to holistically improve patient outcomes.

In the MIYABI ND-C trial, the molidustat group demonstrated significant improvements from baseline in all domain scores at week 24, whereas notable improvements in effectiveness and side effect domain scores were observed in the darbepoetin group. Upon further investigation, there was a significant difference in LS mean changes, as evaluated with a GLM, between molidustat and darbepoetin alfa in the convenience domain at week 12, and a significant difference for this domain was also observed in the GEE analysis in that trial. Enhanced improvement in the convenience domain with molidustat could be attributed to the different routes of administration of molidustat (daily oral tablets) and darbepoetin alfa (SC injection at a hospital). In the MIYABI ND-M trial of patients who received ESAs prior to enrollment, global satisfaction and convenience improved more with molidustat. However, given the small sample sizes, improvements observed with darbepoetin alpha did not reach significance in any domain. In the GLM and GEE analyses, an impact of molidustat on the convenience domain was found at all follow-up time points. It cannot be ruled out that the considerable differences in the frequency and route of administration impacted patient convenience with these treatments.

A preference among patients for oral administration over subcutaneous injection has been demonstrated in other disease areas with blood-related complications, such as long-term treatment of myelodysplastic syndrome (MDS) [19]. In a cross-sectional study, most patients with MDS reported treatment-related interference with social and daily activities as well as pain related to IV/SC treatment administration. Almost 70% of the patients in that study reported that they would prefer oral medication over IV/SC treatment. However, the results of the current post-hoc analysis are not directly comparable to those of observational studies because the trial participants could not be asked about their preference for other possible treatments.

It is also possible that patients found treatment schedules to be more convenient for molidustat. Patients taking molidustat required dose-control visits once every 4 weeks, whereas dose-control visits for darbepoetin alfa users were required every 2 weeks until Hb levels stabilized in the target range as per the study protocol. Furthermore, the frequency of darbepoetin alfa administration and dosage were changed from once every 2 weeks to once every 4 weeks when Hb levels were considered stable. A clinical trial focused on treatment satisfaction for people living with HIV requiring long-term treatment management found that patients preferred less frequent treatment and concluded that the mode of administration is one characteristic that accounts for improved treatment satisfaction [20].

Other domains of the TSQM-II showed remarkable improvement in both trials, albeit less consistently than changes in the convenience domain. Both studies showed better outcomes for perceived effectiveness, in the form of absolute value increases, at weeks 12 and 24 for both the molidustat and darbepoetin groups. These increments were nominally significant for both treatments in patients who had not previously received ESAs. Previous structural equation modeling of responses to the TSQM-II found that the effectiveness and convenience domains contributed significantly to global satisfaction among patients using generic medication [21]. While the association between domains was not examined in the current study, the effectiveness and convenience of molidustat may have had some interaction with the significantly increased global satisfaction domain scores at weeks 12 and 24 in both trials for patients treated with molidustat.

Although this study had several strengths, several limitations should be noted. First, this post-hoc analysis of data from multiple trials did not aim to statistically compare the outcomes of each trial. Furthermore, since both the MIYABI-ND-C and ND-M were open-label, the participants to the trials might be more interested in a HIF-PH inhibitor as a newer treatment compared to an ESA. There was a concern that the open-label design of the study might have introduced bias and partially confounded the outcomes of the trial, but as TSQM data were collected up to week 24th (half of the study period), there were no significant dropouts for both treatment groups. Descriptive comparisons were complicated by slightly different inclusion criteria and patient characteristics. While they are not directly comparable, regardless of prior ESA usage, patients who received molidustat reported higher satisfaction in terms of convenience at the earlier evaluation time point compared with those who received darbepoetin alfa. Overall, perceptions of convenience and global satisfaction with their treatment significantly improved for patients who received molidustat, whereas no significant improvement was seen in those who received darbepoetin alfa. Second, the duration of this study was 24 weeks; therefore, studies with a longer study period are warranted to ascertain long-term effects. Owing to the chronic nature of complications and the long-term treatment for CKD-related anemia, treatment satisfaction may be a key differentiator for patients with CKD-related anemia and their healthcare providers.

## Supplementary Information

Below is the link to the electronic supplementary material.

Supplementary file1 (DOC 29 KB)

## Data Availability

The data that support the findings of this study are available from Bayer Yakuhin, Ltd. but restrictions apply to the availability of these data, which were used under licence for the current study and so are not publicly available. The data are, however, available from the authors upon reasonable request and with the permission of Bayer Yakuhin, Ltd.
