# Supplementary material for: Treatment satisfaction with molidustat in CKD-related anemia in non-dialysis patients: a post-hoc analysis of two clinical trials
Source: Clin Exp Nephrol. 2023 Apr 24;27(8):651–9. doi: 10.1007/s10157-023-02353-x (PMC10352152; doi:10.1007/s10157-023-02353-x)
Supplement: Supplementary file 1 — Supplementary file1 (DOC 29 KB) [file 10157_2023_2353_MOESM1_ESM.doc]

**Title:** Treatment satisfaction with molidustat in CKD-related anemia in non-dialysis patients: a post-hoc analysis of two trials

**Journal name:** Clinical and Experimental Nephrology

Hiroyasu Yamamoto^1^, MD, PhD, ORCID: 0000-0002-5169-0350, Takashi Yamada^2^, MD, PhD, Ken Miyazaki^2^, MEng, Takuto Yamashita^2^, PhD, Takuya Kato^2^, PhD, Kenichi Ohara^3*^, MPH, Yusuke Nakamura^3^, MBA, MPH, Yoko Sakai^4^, MA, Bruce Crawford^4^, MA, MPH, Tadao Akizawa^5^, MD, ORCID: 0000-0002-6150-9201

**Affiliation**

^1^ Division of Nephrology and Hypertension, Department of Internal Medicine, The Jikei University School of Medicine: 3-25-8 Nishi-Shimbashi, Minato-ku, Tokyo 105-8461, Japan

^2^ Research and Development, Bayer Yakuhin Ltd, 2-4-9 Umeda, Kita-ku, Osaka 530-0001, Japan

^3^ Market Access, Bayer Yakuhin Ltd, 1-6-5 Marunouchi, Chiyoda-ku, Tokyo 100-8265, Japan

^4^ Syneos Health, 2-1-3, Nihonbashi, Chuo-ku, Tokyo 103-0027, Japan

^5^ Showa University School of Medicine, Tokyo, 1-5-8 Hatanodai, Shinagawa-ku, Tokyo 142-8555, Japan

**Corresponding author:**

Kenichi Ohara

Mailing address: Marunouchi Kitaguchi Bldg. 1-6-5, Marunouchi, Chiyoda-ku, Tokyo 100-8265, Japan

Email: kenichi.ohara@bayer.com

Telephone number: +81-80-8508-3885

Fax number: +81-3-3282-6713

**Supplementary Table 1** Pearson correlation coefficients for patient characteristics and domain scores at week 24 (MIYABI ND-C).

|  | ND-C | | | | | | | |
| --- | --- | --- | --- | --- | --- | --- | --- | --- |
|  | **Effectiveness**  **(n=95)** | | **Side effects**  **(n=95)** | | **Convenience**  **(n=95)** | | **Global satisfaction**  **(b=95)** | |
|  | r | p-value | r | p-value | r | p-value | r | p-value |
| Hb level at week 24 | 0.052 | 0.615 | 0.073 | 0.482 | 0.029 | 0.784 | -0.048 | 0.647 |
| Average drug dose | -0.183 | 0.076 | 0.098 | 0.342 | -0.029 | 0.777 | -0.124 | 0.231 |

Note: Patients categorized as having high satisfaction at week 24 were included.

MIYABI: Molidustat once daily improved renal anemia by inducing EPO; ND-C: non-dialysis correction

**Supplementary Table 2** Pearson correlation coefficients for patient characteristics and domain scores at week 24 (MIYABI ND-M)

|  | ND-M | | | | | | | |
| --- | --- | --- | --- | --- | --- | --- | --- | --- |
|  | **Effectiveness**  **(n=99)** | | **Side effects**  **(n=99)** | | **Convenience**  **(n=99)** | | **Global satisfaction**  **(b=99)** | |
|  | r | p-value | r | p-value | r | p-value | r | p-value |
| Hb level at week 24 | -0.012 | 0.902 | -0.012 | 0.903 | -0.079 | 0.436 | 0.067 | 0.508 |
| Average drug dose | -0.270 | 0.007* | -0.065 | 0.523 | -0.033 | 0.743 | -0.111 | 0.276 |

Note: * p<0.05; Patients categorized as highly satisfied at week 24 were included.

MIYABI: Molidustat once daily improved renal anemia by inducing EPO; ND-C: non-dialysis correction
